# Investigation of Surface Plasmon Resonance (SPR) in MoS_2_- and WS_2_-Protected Titanium Side-Polished Optical Fiber as a Humidity Sensor

**DOI:** 10.3390/mi10070465

**Published:** 2019-07-11

**Authors:** Rozalina Zakaria, Nur Aina’a Mardhiah Zainuddin, Tan Chee Leong, Rosnadiya Rosli, Muhammad Farid Rusdi, Sulaiman Wadi Harun, Iraj Sadegh Amiri

**Affiliations:** 1 Photonic Research Centre, Faculty Science, University of Malaya, 50603 Kuala Lumpur, Malaysia; 2 Department of Electrical Engineering, Faculty of Engineering, University of Malaya, 50603 Kuala Lumpur, Malaysia; 3 Computational Optics Research Group, Advanced Institute of Materials Science, Ton Duc Thang University, Ho Chi Minh City 700000, Vietnam; 4 Faculty of Applied Sciences, Ton Duc Thang University, Ho Chi Minh City 700000, Vietnam

**Keywords:** side-polished fiber (SPF), molybdenum disulfide (MoS_2_), tungsten disulfide (WS_2_)

## Abstract

In this paper, we report the effects of a side-polished fiber (SPF) coated with titanium (Ti) films in different thicknesses, namely 5 nm, 13 nm, and 36 nm, protected by a thin layer of transition metal dichalcogenides (TMDCs) such as molybdenum disulfide (MoS_2_) and tungsten disulfide (WS_2_), which provide ultra-sensitive sensor-based surface plasmon resonance (SPR) covering from the visible to mid-infrared region. The SPF deposited with Ti exhibits strong evanescent field interaction with the MoS_2_ and WS_2_, and good optical absorption, hence resulting in high-sensitivity performance. Incremental increases in the thickness of the Ti layer contribute to the enhancement of the intensity of transmission with redshift and broad spectra. The findings show that the optimum thickness of Ti with 36 nm combined with MoS_2_ causes weak redshifts of the longitudinal localized surface plasmon resonance (LSPR) mode, while the same thickness of Ti with WS_2_ causes large blueshifts. The redshifts are possibly due to a reduced plasmon-coupling effect with the excitonic region of MoS_2_. The observed blueshifts of the LSPR peak position are possibly due to surface modification between WS_2_ and Ti. Changing the relative humidity from 58% to 88% only elicited a response in Ti/MoS_2_. Thus, MoS_2_ shows more sensitivity on 36-nm thickness of Ti compared with WS_2_. Therefore, the proposed fiber-optic sensor with integration of 2D materials is capable of measuring humidity in any environment.

## 1. Introduction 

**Figure 1:**
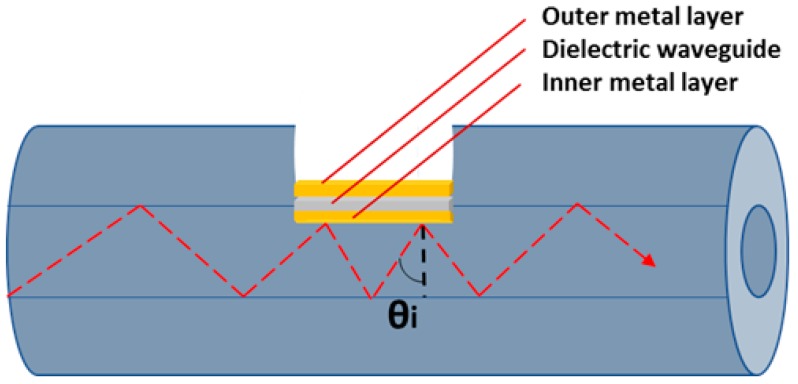
Schematic diagram of light propagation inside the optical fiber.

Surface plasmon resonance (SPR) is a well-known optical phenomenon that occurs on a metal-dielectric surface. When free electrons oscillate on the metal surface, the surface absorbs light energy and resonates during the reflection of light at the SPR angle, which has a major attenuation effect on reflected light, as shown in Figure 1 [1]. SPR is highly sensitive to the refractive index of the material in contact with the metal, which can be used as a sensing structure such as the Kretschmann Rather prism, optical waveguides, and optical fiber [2,3,4,5]. SPR is also used as an optical phenomenon to detect molecular interaction [6].

The properties of metallic layers [7] in various environments confer SPR properties that have their own benefits, such as the fact that a beam of light cannot pass through it (it can be absorbed in a dielectric medium), and diffusion of light inside an analyte [8]. Thus, SPR is potentially focused on the characteristics of the metal and the dielectric medium layer. Titanium (Ti) has excellent characteristics, such as high corrosion resistance, and high-adhesion layer and passivation surface, which are enough properties to construct an SPR-sensor [9,10]. There are several techniques to achieve nanomaterials-based optical sensors, such as the deposition of a thin film on a fiber core, microfiber, and side-polished optical fiber [11,12,13]. 

The practical use of side-polished fiber has several advantages due to compactness, simple fabrication method, and low insertion loss [14]. Besides that, the use of dielectric material coating on Ti film enhances their electrical and optical properties externally. Ti in rutile forms exhibits high dielectric constant, which makes Ti more useful for sensing applications. Some features such as high surface-to-volume ratio and tunable biocompatibility are some of the factors that can enhance the sensitivity in the device. The use of Ti coating also protects the metal from oxidation [15]. Using the side-polishing technique, a single-mode fiber (SMF) can be fabricated, as the technique removes a portion of the cladding region [16,17,18]. By doing so, an evanescent field can be created whereby light escapes from the core region and is propagated within the polished surface region. The interaction of the evanescent field with the external environment forms the basis of an SPR sensor [19,20]. Using this technique, many kinds of sensors, including devices based on optical fiber sensors, can be fabricated and used in many applications such as ultraviolet (UV) power sensors, polarization controllers, and all-fiber integrated optical power monitors [21]. 

The side-polished technique was used in this experiment to increase sensitivity where the propagating light inside the core directly reflects or beams onto the metal-dielectric layer. The integration of several nanomaterials in the development of various optical sensors, such as TiO_2_ [22,23] in optical temperature sensors and alcohol sensors, reduced graphene oxide [24] as well as graphene [25,26]. Graphene has been explored widely; however, it suffers from the gapless of band-structure properties. Hitherto, several semiconducting two-dimensional materials have been recognized as excellent candidates for sensing devices in the future. Molybdenum disulfide (MoS2) is a suitable material due to its various optical characteristics such as temperature sensing [27,28,29,30], including various technological applications using WS_2_ [31,32]. Here, we investigated the optimum Ti thickness to provide SPR behavior with an integration layer of MoS_2_ and WS_2_ in a relative humidity (RH) sensing application in the visible to mid-infrared region.

## 2. Experimental

**Figure 2:**
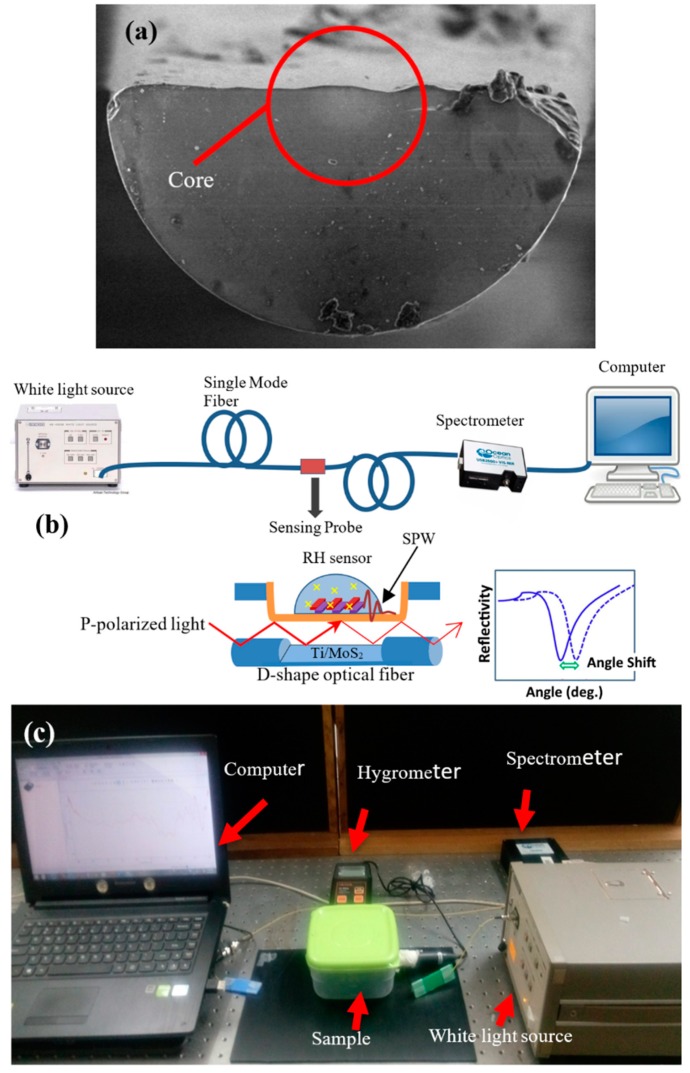
(**a**) Field emission scanning electron microscopy (FESEM) image of side-polished single-mode fiber (SMF). (**b**) Experimental diagram of Ti/MoS_2_/WS_2_ RH sensor; SPW = surface plasmon wave. (**c**) Humidity sensor set-up in the laboratory.

The fabrication process of polishing SMF involves using an abrasive polishing wheel. This approach requires the SMF itself with a core/cladding diameter of 9/125 µm fixed on its position by utilizing a pair of fiber holders, where a small section of coating on SMF has been stripped off. The mechanical wheel was fastened by the abrasive paper in order to control the smoothness of the polished region. This process, which tends to have a success rate close to 100%, observably reduces the polishing time and provides a uniform polished active region. Here, the actual sensing region of the SMF was a 3-mm flat transition region, as shown in the image obtained by field emission scanning electron microscopy (FESEM) in Figure 2a. SMF cladding was removed, and the loss of the fiber was recorded as -2dB during the polishing process. A broadband light source with a range of 900 nm to 1500 nm and a spectrometer were used to measure the transmission attenuation of the SPF during the experiment. The polished portion of the flat surface was coated with 5-nm, 13-nm, and 36-nm thicknesses of Ti using an electron beam evaporation machine, followed by drop cast of MoS_2_ and WS_2_ layer of about 0.2 mL. A thin layer of Ti was deposited on flat-clad less optical fiber using an electron beam evaporation machine (EB43-T) at a high vacuum pressure of 10^−5^–10^-7^ Torr. Then, the sensitivity was recorded using a humidity sensor device with different relative humidity values (RH%). At the same time, the light of the polychromatic source was launched from one end of the fiber, and the other end of the fiber was connected to an ocean optic spectrometer. The output of the transmission spectra was recorded. A schematic diagram and a real image of the experimental set-up are shown in Figure 2b,c. The set-up for characterizing the resonance peak for TiO_2_/MoS_2_ and WS_2_ is shown in Figure 2b,c, in which one end of the fiber was coupled to a white light source, and the output was connected to AQ6370B Optical Spectrum Analyzer (Yokogawa Electric Corporation, Tokyo, Japan) with a spectral resolution of 2 nm with a range of 700 nm to 1800 nm.

## 3. Results and Discussion

### 3.1. SPR-Transmission with Different Thicknesses of Titanium

**Figure 3:**
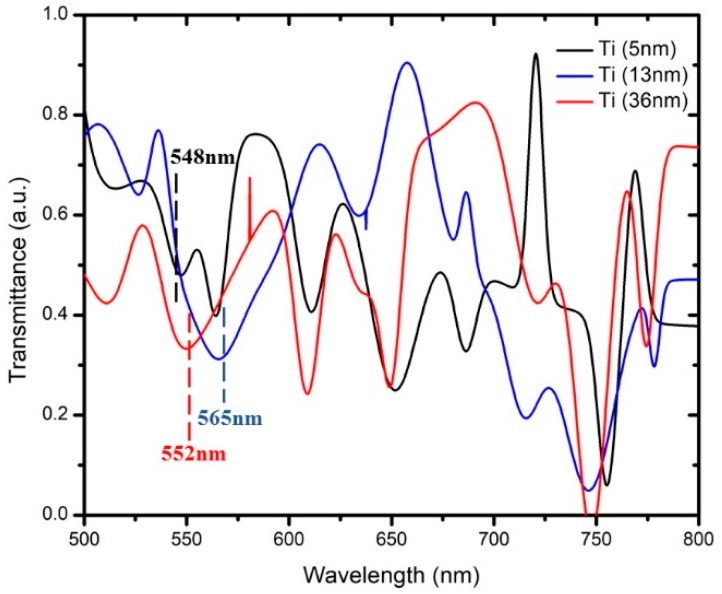
Dip of transmission based on surface plasmon resonance (SPR) with different thicknesses of titanium set for MoS_2._

**Figure 4:**
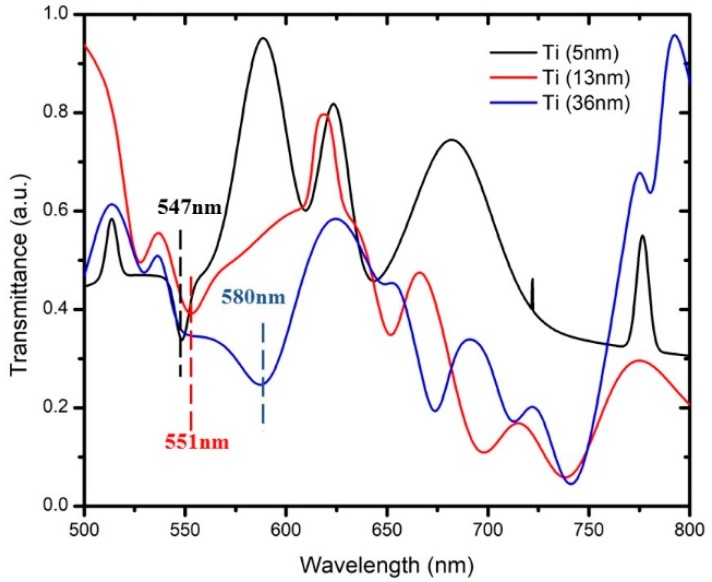
Dip of transmission based on SPR with different thicknesses of titanium set for WS_2_.

Figure 3 and Figure 4 show the transmission spectrum of wavelength at different thicknesses for MoS_2_ and WS_2_, respectively. There were six samples that were coated with Ti at thicknesses of 5 nm, 13 nm, and 36 nm (two samples each). Samples with the same Ti thickness were tested with a layer of 2D materials: MoS_2_ or WS_2_. The effectiveness of these 2D materials was compared with regards to other thicknesses of Ti coating. The SPR for Ti was recorded at the range of 500 nm to 600 nm [16]. Based on the SPR of Ti, this range of wavelength will contribute to the most sensitive shifting due to the optimum thickness. The detection of SPR phenomena can be observed with respect to the wavelength corresponding to the transmission dip in the spectrum. The transmission dip is formed where the reflected light resonates onto the surface plasmon after it passes through the SPR region. In this work, it was determined at the other end of the optical fiber as a function of wavelength [9]. Figure 3 shows that the transmission dip for different thicknesses of Ti at 5 nm, 13 nm, and 36 nm occurred at wavelengths of 548 nm, 552 nm, and 565 nm, respectively. These three samples were tested with a monolayer of MoS_2_ in aqueous states. Figure 4 shows the transmission dip for different thicknesses of Ti with wavelengths of 547 nm, 551 nm, and 580 nm, respectively. These three samples were tested with a monolayer of WS_2_ for sensing performance. Both figures showed that the transmission dip becomes broader with increasing thickness of Ti. 

For example, the Ti thickness of 5 nm has a sharper dip compared with the 13 nm and 36 nm. Based on the SPR phenomena, the number of reflections is a crucial rule that affects the width of the transmission dip in the spectrum [9]. The number of light reflections relies on the length of the sensing area and the diameter of the optic fiber. Sensitivity is explained in the next section, whereby a drop of dielectric medium such as MoS_2_ and WS_2_ was applied to form a humidity sensor. Thus, with a greater thickness of the Ti layer, the transmission-based surface plasmon resonance (SPR) shifts to the right, which increases the wavelength transmission dip. Observing the transmission spectrum, as shown in Figure 3 and Figure 4, we can clearly see that it has a large dip in the constant range of wavelength. The modes were divided into two types: the transverse mode is the oscillation across the width of the optic fiber waveguide, and the longitudinal mode is the oscillation along the length of its cavity. In this experiment, Ti affected the transverse mode of optical-fiber-based SPR according to the characterization from the transmission electron microscope (TEM_00_). The allowed modes can be found by solving Maxwell’s equation for the condition of the waveguide boundary. The dip of transmission for different thickness of Ti was chosen based on the transverse mode.

### 3.2. Effect of TMDCs with Different Thicknesses of Titanium

**Figure 5:**
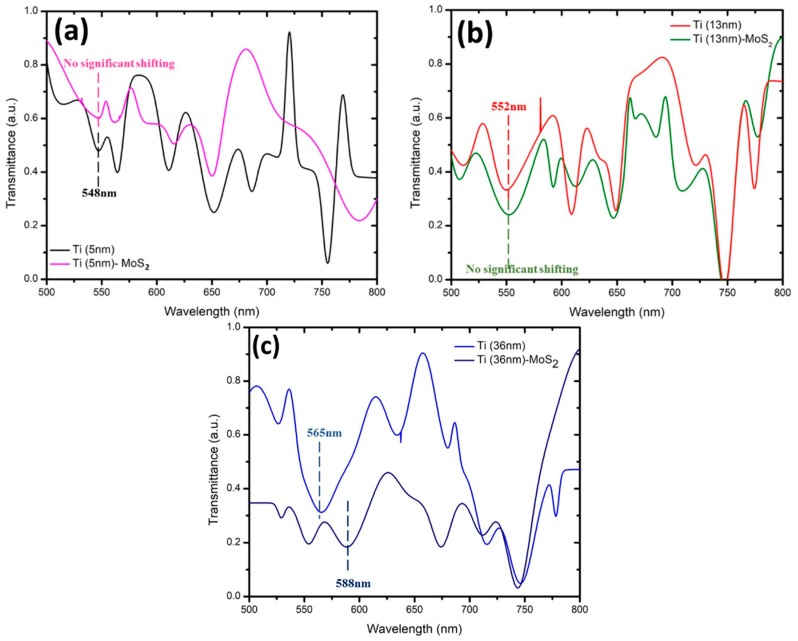
Dip of transmission based on SPR with (**a**) 5-nm, (**b**) 13-nm, and (**c**) 36-nm thickness of titanium covered with MoS_2._

We observed that no significant shifting occurred at the fixed or constant range of wavelength for a thinner layer of Ti (Figure 5a,b). This indicates that the volume of 0.2 mL was not suitable with 5-nm and 13-nm thicknesses of Ti. In Figure 5c, the transmission dip for 36 nm of Ti layer shows a positive response to the 0.2 mL of MoS_2_ with an initial transmission wavelength of 565 nm shifting to the right to about 588 nm, which shows low frequency at a higher wavelength. The range of transmission response recorded was about 23 nm, where the 36-nm thickness of Ti was found to be suitable for use as a sensor with 0.2 mL of MoS_2_. The response of MoS_2_ to the different thicknesses of Ti is due to characteristics such as the thickness of adhesion on the surface of Ti, which is related to the roughness of the surface. For example, Ti has high corrosion resistance and a high-adhesion layer that forms a passivation surface when exposed to surrounding air. It has also been reported that the properties of the Ti layer do not change, and it is less absorptive compared to titanium alloys. Moreover, it is good in the phase of detection. In this case, the adhesion layer of Ti plays important roles in the composition of Ti/MoS_2_. MoS_2_ also has a low shear strength of its basal planes, with a friction coefficient as low as 0.01 in a vacuum environment. Furthermore, the coating of MoS_2_ has low resistance and can react with O and H_2_O in an atmosphere of high humidity to form MoO_3_ and H_2_SO_4_. Both of these compounds can lead to the corrosion of metal and degradation of coatings, and the low-adhesion layer of MoS_2_ is covered by the high adhesion layer of Ti. Based on the shift described for 36-nm thickness of Ti, the increased thickness of Ti provides an increased adhesion layer that reduces the rough surface of the Ti/MoS_2_ layer. 

**Figure 6:**
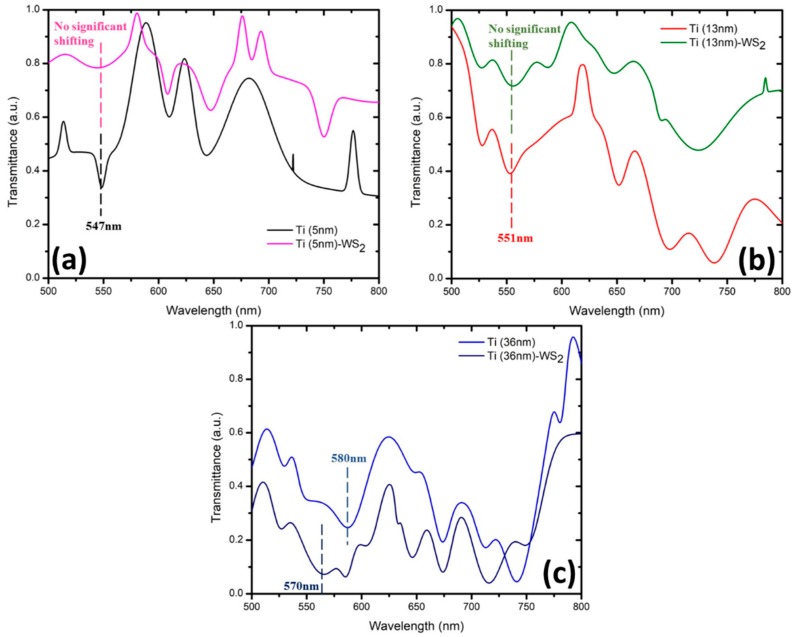
Dip of transmission based on SPR with (**a**) 5-nm, (**b**) 13-nm, and (**c**) 36-nm thickness of titanium by covered with WS_2_.

Figure 6 shows the same phenomena with MoS_2_, where we observed no shifting occurring at the fixed or constant range of wavelength. Both transmission dips at 570 nm and 580 nm do not give a response with a thinner layer of Ti. This indicates that the volume of 0.2 mL of WS_2_ is not enough to provide sensor properties to the Ti thickness shown in Figure 6a,b. In Figure 6c, the transmission dip for the 36-nm Ti layer showed a response to the 0.2 mL of tungsten disulfide (WS_2_), whereby the transmission dip at 580 nm shifted to the left to about 570 nm. The left shifting is known as blueshift where it has a higher frequency at a lower wavelength. The range of transmission response was large, about -15nm. The negative sign is functional, as the transmission wavelength is reduced. Due to the response of shifting, the 36-nm thickness of Ti appears suitable with 0.2 mL of WS_2_.

From Figure 5 and Figure 6 we observe some differences in shifting. For example, right shifting (redshift) and left shifting (blueshift) are occurring for the 36-nm thickness of Ti when coated with MoS_2_ and WS_2_, respectively. The shifting whether left (blueshift) or right (redshift) is due to the shape and size resonance frequency of the particle of material. The red shifting occurs based on the increase in the dielectric constant that surrounds the medium where it has low frequency and high wavelength. Besides that, the formation of blueshifts happens according to the decreasing particle size where it has high frequency and low wavelength. The frequency is inversely proportional to the wavelength. The shape character tends to become more complex while the orientation of particles of the medium or material leads to additional resonance [20]. Therefore, the polarization of the medium also provides another way to observe the SPR shift, besides the shape and size of the particles. A greater polarization of the medium is created due to the increase in dielectric function by a higher refractive index. The greater polarization of the medium will reduce the collective charge in the resonance phase. The attenuation of the particle electric charge tends to reduce the electric force that will reduce the resonance frequency, and the transmission dip will be redshifted. This phenomenon can be verified by using the simulation of Mie theory. It is the surrounding medium that determines the resonance frequency of a particle besides its size and shape. In case of metallic particles, the frequency of the localized surface plasmon resonance (LSPR) is always redshifted compared to the plasma frequency. A decrease in size leads to a blueshift (higher frequency), while an increase in the dielectric constant of the surrounding medium leads to a redshift (lower frequency). The influence of shape can be very complex and lead to additional resonances (e.g., in the case of cubes) and/or dependence on the orientation of the particles (e.g., in cases of ellipsoids). The spectrum featured a slight blueshift as a contribution emission of WS_2_ due to the arisen LSPR, as reported by Choi et al. [33]. A model dispersion of SPR was successfully modeled and reported by Yuk Sun Jung et al. [34], where a metal of finite extent and juxtaposed with a dielectric can be supported at the metal/dielectric interface. Surface plasmons have a divergence-free (∇·D = 0) transverse electromagnetic mode associated with charge density oscillations at the interface. The resonance condition of this surface-bound wave is sensitive to the size and geometry of the metal and its surrounding dielectric. Where here, in the case of a planar metal/dielectric interface, surface plasmons (SPs) experience a medium with an effective dielectric constant, ε_m_ε_d_/(ε_m_ + ε_d_), and the surface plasmon resonance frequency ω_sp_ is determined from the condition, ε_m_ (ω_sp_) + ε_d_ (ω_sp_) = 0. The presence of the dielectric material causes the SP resonance frequency (ω_sp_) to be redshifted from that of the bulk plasmon frequency (ωp). The consequence of blueshifts is due to surface modification and interaction between layer of WS_2_ and Ti where it intrinsically distributed resonant cavity of (SP) dispersion.

### 3.3. Sensitivity in Relation to Relative Humidity (RH%)

#### Sensitivity of Best Thickness of Ti with MoS_2_

**Figure 7:**
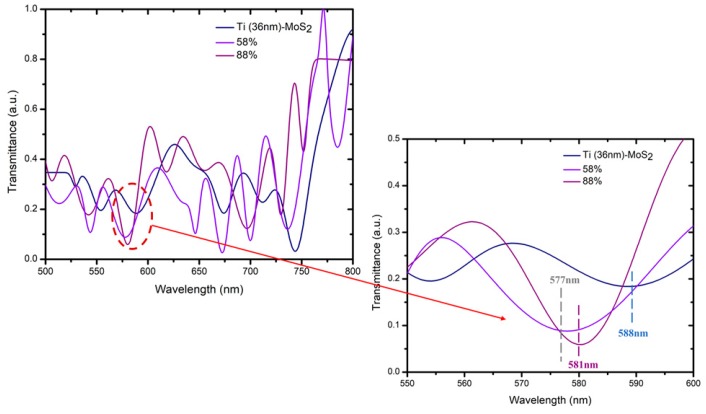
Sensitivity of 36-nm thickness of titanium covered by MoS_2_, Ti (36-nm)/MoS_2_.

**Figure 8:**
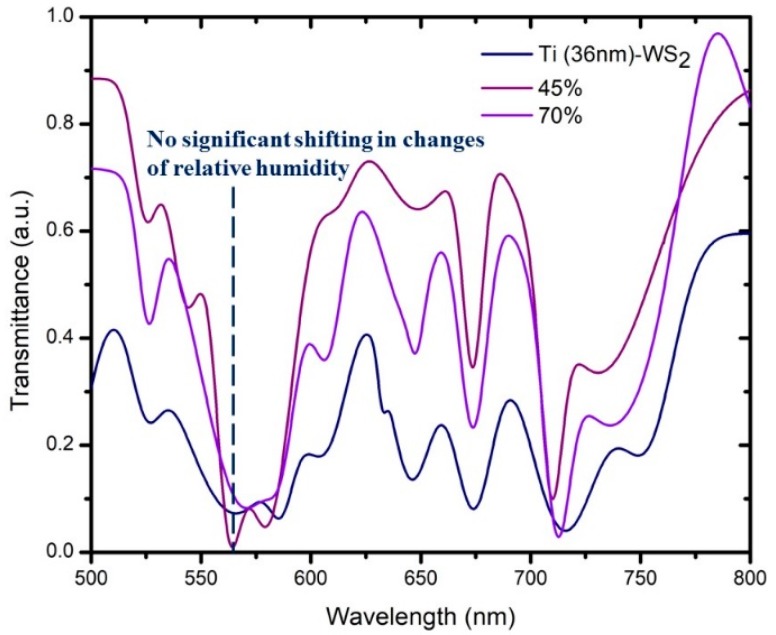
Sensitivity of 36-nm thickness of titanium covered by WS_2_, Ti (36-nm)/WS_2_.

In Figure 7 and Figure 8, we have simplified by selecting only 36-nm Ti due to its response when tested with MoS_2_ and WS_2_. Figure 7 shows that with MoS_2_, there was a change in the shift of the transmission dip when the relative humidity changed from 58% to 88%. The transmission dips for 58% and 88% of relative humidity were at wavelengths of 577 nm and 581 nm, respectively. Therefore, MoS_2_ appears to be highly sensitive toward 36-nm titanium in a constant range of transmission wavelength. The selection of the transmission dip in the humidity sensor was based on the similar shape of the transmission dip in the spectrum of Ti-MoS_2_ (36 nm) at 588 nm. Figure 8 shows the spectra with WS_2_ when the RH% changes from 45% to 70% as there is no shift of the transmission dip. Thus, it appears that WS_2_ was not as sensitive toward 36-nm titanium in the constant range of transmission wavelength even in higher RH%. The selection of transmission dip in the humidity sensor was based on the similar shape of the transmission dip for Ti-WS_2_. The sensitivity of the humidity sensor can be evaluated by the range of relative power based on the optical characteristics divided by the range of relative humidity (∆RP/∆RH) [16]. However, in this case, the sensitivity was determined by observing the response of shifting in the transmission dip. Therefore, compared with WS_2_, MoS_2_ was more sensitive when reacting with titanium.

## 4. Conclusions

In conclusion, the range of surface plasmon resonance for titanium was around 500–600 nm. The selection of the transmission dip of different thickness in terms of surface plasmon resonance was based on the transverse mode related to the Maxwell equation. The shifting dip is then observed with the same shape of dip selected with the first sample. Higher thickness of Ti coating results in increased wavelength of the transmission dip with broadening in the spectrum. Both thickness of 5 nm and 13 nm of titanium did not show a reaction when applying the MoS_2_ or WS_2_ layer. Only the 36-nm titanium reacted when coated with MoS_2_ and WS_2_ due to the adhesion layer of titanium and structure of the dielectric medium (MoS_2_ or WS_2_). Thus, the best thickness of titanium when coated by either MoS_2_ or WS_2_ was 36 nm. These shift in different directions, with Ti (36-nm)/MoS_2_ and Ti (36-nm)/WS_2_ shifting to the right (redshift) and left (blueshift), respectively. The redshift has low frequency, while the blueshift has high frequency, with the frequency being inversely proportional to the wavelength. The size and shape of the particle affected the resonance and produced the direction of shifting. Therefore, we simplified the detection of sensitivity using a 36-nm thickness of the titanium layer, as this was the only one showing shifting. The sensitivity was determined by observing the shifting of the transmission dip due to the changes in relative humidity (RH%). The selection of the transmission dip in humidity sensing was based on a similar shape as the transmission dip of Ti(36-nm)/MoS_2_ or Ti(36-nm)/WS_2_. MoS_2_ was more sensitive to the Ti layer compared with WS_2_.
